# Ultrasound screening for asymptomatic carotid stenosis in subjects with calcifications in the area of the carotid arteries on panoramic radiographs: a cross-sectional study

**DOI:** 10.1186/1471-2261-11-44

**Published:** 2011-07-13

**Authors:** Elias P Johansson, Jan Ahlqvist, Maria Garoff, Kjell Karp, Eva Levring Jäghagen, Per Wester

**Affiliations:** 1 Department of Public Health and Clinical Medicine, Umeå University, Umeå, Sweden; 2 Department of Odontology, Umeå University, Umeå, Sweden; 3 Clinical physiology, Department of Surgical and Perioperative Sciences, Umeå University, Umeå, Sweden

## Abstract

**Background:**

Directed ultrasonic screening for carotid stenosis is cost-effective in populations with > 5% prevalence of the diagnosis. Occasionally, calcifications in the area of the carotid arteries are incidentally detected on odontological panoramic radiographs. We aimed to determine if directed screening for carotid stenosis with ultrasound is indicated in individuals with such calcifications.

**Methods:**

This was a cross-sectional study. Carotid ultrasound examinations were performed on consecutive persons, with findings of calcifications in the area of the carotid arteries on panoramic radiography that were otherwise eligible for asymptomatic carotid endarterectomy.

**Results:**

Calcification in the area of the carotid arteries was seen in 176 of 1182 persons undergoing panoramic radiography. Of these, 117 fulfilled the inclusion criterion and were examined with carotid ultrasound. Eight persons (6.8%; 95% CI 2.2-11.5%) had a carotid stenosis - not significant over the 5% pre-specified threshold (p = 0.232, Binomial test). However, there was a significant sex difference (p = 0.008), as all stenoses were found in men. Among men, 12.5% (95%CI 4.2-20.8%) had carotid stenosis - significantly over the 5% pre-specified threshold (p = 0.014, Binomial test).

**Conclusions:**

The incidental finding of calcification in the area of the carotid arteries on panoramic radiographs should be followed up with carotid screening in men that are otherwise eligible for asymptomatic carotid endarterectomy.

**Trial Registration**

The study was registered at http://www.clinicaltrials.gov; NCT00514644

## Background

In patients with asymptomatic carotid stenosis, carotid endarterectomy (CEA) reduces the net risk of stroke and perioperative events at 5 [1] and 10 years follow-up [2]. Patients ≥ 75 years of age do not benefit from asymptomatic CEA [2]. The benefit of asymptomatic CEA has come into question since a lower risk of stroke without CEA has been shown in recent observational studies compared to the randomized studies [3]. This can, at least in part, be explained by that lipid lowering medications were less commonly used during the time period of these randomized trials [3]. Current guidelines suggest that asymptomatic CEA should only be performed when the perioperative risk is low [4-6]. One ongoing randomized study will determine if patients with statin and other cardiovascular preventive treatment benefit from asymptomatic CEA [7]. There is limited but promising evidence that improved patient selection to asymptomatic CEA can be achieved by plaque characteristics, microemboli detection and cerebrovascular reactivity testing [8-10].

In a systematic review, the prevalence of asymptomatic carotid stenosis was 7.5% in men aged ≥ 80 years, 5.0% in women aged ≥ 80 years, 2.3% in men aged 60-69 years and 2.0% in women aged 60-69 years [11]. Asymptomatic carotid stenosis can be detected incidentally - e.g. detection of a contralateral lesion when examining patients after a transient ischemic attack (TIA) or stroke - or by directed screening. Directed screening for asymptomatic carotid stenosis is suggested to be cost-effective in populations with > 5% prevalence and low perioperative risk, and in populations with > 20% prevalence of carotid stenosis and moderate perioperative risk [12].

**Figure 1:**
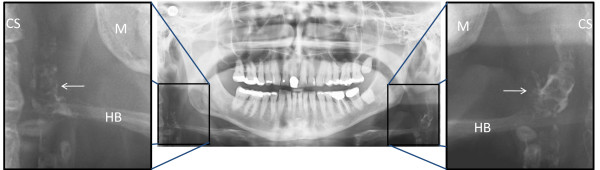
**Panoramic image**. Showing bilateral calcification in the area of the carotid arteries (arrows). (M = mandible, CS = cervical spine, HB = hyoid bone).

In practice, panoramic radiographs are performed prior to routine dental care, implant placement, trauma, and local cancer treatment. In 3.5-4.2% of these persons, a calcification in the area of the carotid artery is detected [13,14] (see Figure 1). A calcification in the area of the carotid arteries might indicate a carotid stenosis, since the calcification could be part of an atherosclerotic plaque that reduces the lumen more than 50%. Some carotid stenoses contain calcifications while others do not. In two previous studies, 85 individuals with calcifications in the area of the carotid artery were examined with carotid ultrasound; 50-99% carotid stenosis was detected in 26% of the corresponding carotid arteries [13,14].

The aim of this study is to determine if screening with carotid ultrasound is indicated in persons, otherwise eligible for asymptomatic CEA, with calcification in the area of the carotid arteries incidentally detected on panoramic examinations. Screening is considered indicated if the prevalence of stenosis exceeds 5%.

## Methods

### Study group

We interpreted 1182 consecutive panoramic examinations for calcifications in the area of the carotid arteries in a prospective manner. Persons < 18 or ≥ 75 years are not eligible for asymptomatic CEA and were not included [1,2]. Examinations were performed at the department of Oral and Maxillofacial Radiology, Umeå, Sweden, between August 1^st ^2007 and February 26^th ^2009. Age, sex, and indication for the examination were recorded for all persons.

**Figure 2:**
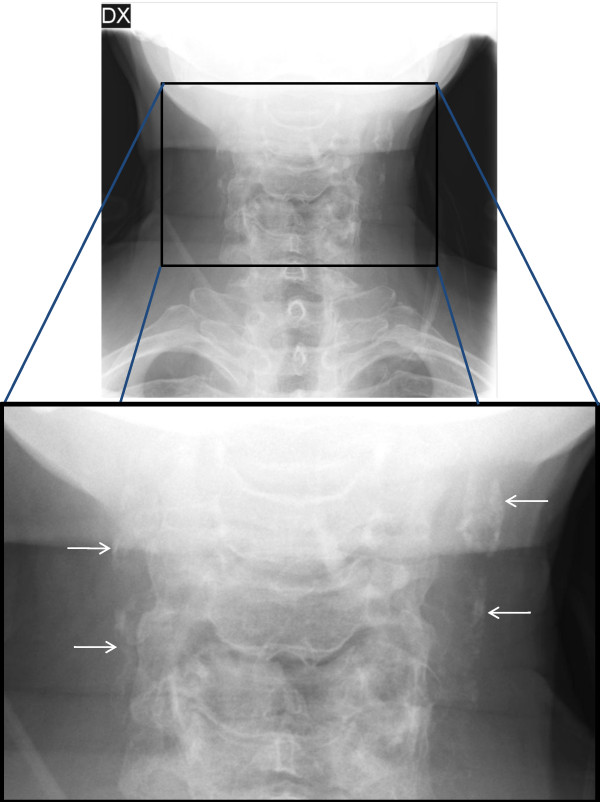
**Anterio-posterior projection of the neck**. Showing calcifications in the area of the carotid arteries (arrows) adjacent to the cervical spine.

**Figure 3:**
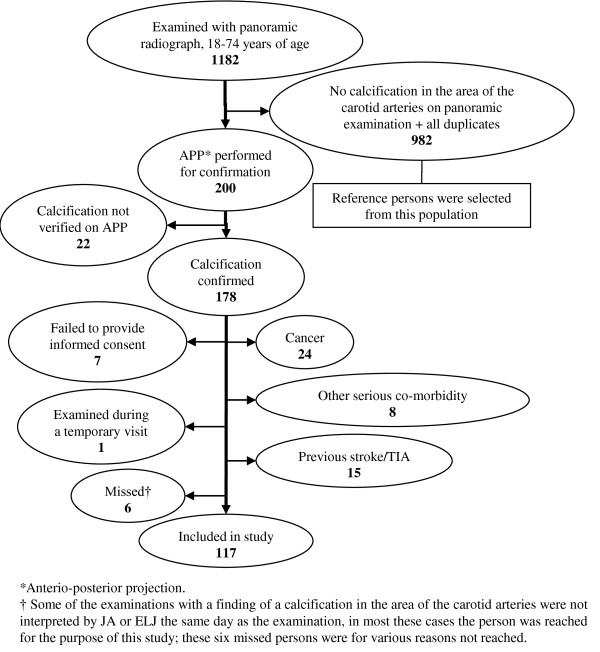
**Trial profile**.

When calcification in the area of the carotid arteries was detected on a panoramic radiograph the radiographic examination was extended with an anterio-posterior projection (APP) of the neck. If the APP confirmed the calcification the individual was included in the study if they were otherwise eligible for asymptomatic CEA. We excluded persons with cancer or other serious co-morbidities whom were not eligible for asymptomatic CEA due to a short life expectancy and/or increased perioperative risk. We excluded all persons with a previous stroke or TIA since we aimed to study persons without any pervious cerebrovascular event. Refer to Figure 1 and 2 for an example of a calcification in the carotid arteries detected on a panoramic radiograph and confirmed on an APP. Medical records were reviewed of all participants, see Figure 3 for trial profile.

### Reference group

**Figure 4:**
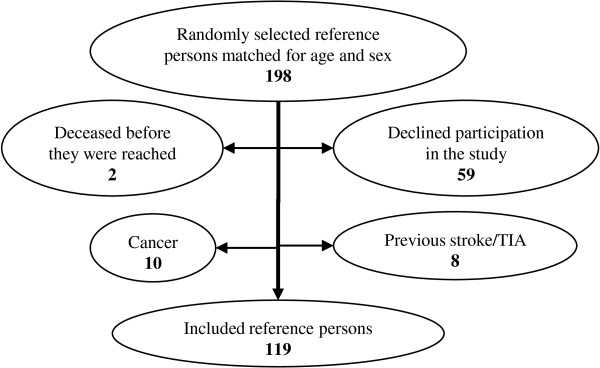
**Reasons for reference persons to be excluded from the study**.

We selected a sex- and age-matched reference group in order to determine if persons with calcification in the area of the carotid arteries have an average or above average burden of atherosclerotic disease. The reference participants were recruited among the persons in whom the panoramic examination did not reveal any calcification in the area of the carotid arteries (Figure 3). One hundred ninety-eight reference persons were randomly selected. Their medical background was assessed via questionnaire; 79 were excluded based on questionnaire results (Figure 4). The reference population did not undergo carotid ultrasound.

### Subgroups

Pre-specified subgroups were based on sex, age, and the cause of referral for the panoramic radiograph examination. With all persons ≥ 75 years excluded, we chose subgroups based on age of < 65 years and 65-74 years since these were the age groups used in ACST-study [1,2]. The cause of referral was divided into three subgroups:

1. Regular dental: Examinations performed in all practices using panoramic radiographs, e.g. dental and implant treatment.

2. Specialised dental: Examinations performed in specialist dental clinics such as fractures and orthodontics.

3. Specialised medical: Examinations routinely performed prior to medical intervention, such as heart valve surgery.

Since there were a low number of included persons in the subgroups "specialised dental" and "specialised medical", these two subgroups were merged for analyses.

Pre-specified cardiovascular subgroups were cardiovascular event as a combined group and for each parameter separately as follows: prior myocardial infarction, current angina pectoris, heart failure, symptomatic peripheral artery disease, or other relevant cardiovascular event. Cardiovascular risk factor was used as a combined group and for each parameter separately as follows: diabetes, hypertension, current smoking, or current usage of antihypertensive medication, lipid lowering medication, or platelet inhibiting or anticoagulant medication.

### Procedures

Panoramic examinations were performed with an Orthopantomograph^® ^OP100, Finland, using the P1-program. APP examinations were performed using a Cranex^® ^Cephalostat, Soredex, Finland, exposed with 81 kV and 10 mA, for 0.8-1.2 s depending on patient size. The APP gives an almost orthogonal projection of the region relative to the panoramic projection and increases the possibility to make a correct interpretation of the position of the calcification [14]. The images were captured with the image plate system, Fujifilm FCR. Fuji IP cassette type cc, size 15 × 30 (panoramic examination) and 18 × 24 cm (APP), respectively, Fuji Photofilm Co., LTD, Japan, were used and an image reader, Fujifilm FCR Capsula XL, Japan, was used for scanning. All images were interpreted using the CDR^® ^DICOM 3.5 software, Schick, USA in a room allowing ultimate dim light conditions. The screens used were RadiForce R22 diagnostic computer screen from Eizo Nanao Corporation, Japan, or the 19-inch diagnostic LCD monitor from Olórin, Sweden.

Two experienced specialists in oral and maxillofacial radiology (JA, ELJ) separately re-evaluated the panoramic examination and APP of the included persons with calcification in the area of the carotid arteries. The inter-reader kappa was 0.69. For study inclusion/exclusion a consensus decision was reached for all neck sides.

Carotid ultrasound examinations were performed by experienced vascular sonographers blinded to the results of the panoramic examination. A Siemens Acuson Sequoia 512^® ^ultrasound system with an 8L5 linear transducer was in all examinations. A visible plaque detected in B-mode with a maximum systolic velocity in the internal carotid artery of 1.45-2.4 m/s and > 2.4 m/s was considered as 50-69% and as 70-99% carotid stenosis, respectively. Carotid occlusion was diagnosed if there was no detectable flow [15]. These criteria have previously been validated locally [16].

### Statistics

We calculated prevalence with 95% confidence intervals. To determine if the prevalence of carotid stenosis was significant above 5% we used the one-tailed nonparametric binomial test with the exact calculation method; we chose this test for significance over the lower boundary of the 95% confidence interval since the latter has lower statistical power and is only an approximation for binary variables (whereas the binomial test is an exact measurement for binary variables). Significance of differences between various groups was determined with the chi-2-test or t-test; in all analyses of the prevalence of carotid stenosis, the presented subgroups were pre-specified. We pre-selected a p-value < 0.05 as cut-off for all relevant calculations. SPSS version 17.0 was used for all calculations.

The sample size was determined by an interim analysis after inclusion of 100 persons that had undergone carotid ultrasound examination. After the interim analysis the study inclusion was closed. The sample size of reference persons was aimed to include at least as many reference participants as there were non-reference participants.

### Ethical considerations

The Regional Ethical Review Board in Umeå approved this study. The study was registered at http://www.clinicaltrials.gov, NCT00514644 before it was launched. All included and reference persons provided informed consent.

## Results

Calcification in the area of the carotid arteries, seen in panoramic radiographs and confirmed with an APP, was detected in 178 persons. We excluded 61 persons with calcification in the area of the carotid arteries since they either were not eligible for asymptomatic CEA, had a history of stroke or TIA, or were not able to participate in the study (Figure 3). See Table 1 for baseline characteristics.

**Table 1:** Demographic data of all persons undergoing panoramic radiograph examination

Group		n	Included n (%)	Significance*
Sex	Women	557	53 (9.5%)	p = 0.677
	Men	625	64 (10.2%)	
Age (years)	< 65	827	40 (4.8%)	p < 0.0001
	65-74	355	77 (21.7%)	
Indication for	Regular dental	668	94 (14.1%)	p < 0.0001‡
panoramic	Specialised dental	279	6 (2.2%)	
examination†	Specialised medical	235	17 (7.2%)	
All		1182	117 (10.1%)	-

*Chi-2-test.

† Regular dental - examinations performed in all practices using panorama, e.g. dental and implant treatment. Specialised dental - examinations performed in specialist dental clinics such as fractures and orthodontics. Specialised medical - examinations routinely performed prior to medical interventions, e.g. heart valve surgery.

‡ Overall difference between the three groups.

Eight of the included persons with a calcification in the area of the carotid arteries (6.8%, 8/117; 95%CI 2.2-11.5%) had in total nine vessels with 50-99% stenosis - not significantly over the 5% pre-specified threshold, p = 0.232 (binomial test).

We detected subgroup differences in the prevalence of 50-99% carotid stenosis (Table 2). The prevalence was significantly higher among men (12.5%; 95% CI 4.2-20.8%), persons with previous myocardial infarction (16.7%; 95% CI 0.6-32.7%), current angina (23.5%; 95% CI 1.1-46.0%), symptomatic peripheral artery disease (50.0%; 95% CI 0.0-100%), current smokers (19.0%; 95% CI 0.7-37.4%), and in persons taking lipid lowering medication (13.1%; 95% CI 4.4-21.8%), platelet or anticoagulant medication (15.1%; 95% CI 5.1-25.1%), or a history of any cardiovascular event (15.9%; 95% CI 4.7-27.2%). We determined, with the binomial test, that the prevalence of 50-99% carotid stenosis was significant over the 5% pre-specified threshold in the following subgroups: men (p = 0.014), history of myocardial infarction (p = 0.030), current angina (p = 0.009), symptomatic peripheral artery disease (p = 0.014), current smoking (p = 0.019), taking lipid lowering medication (p = 0.011), taking platelet inhibiting or anticoagulant medication (p = 0.005), and history of any cardiovascular event (p = 0.006).

**Table 2:** Factors influencing the prevalence of 50-99% carotid stenosis among included persons

	Yes		No		Significance*
	n	With stenosis n (%) [95%CI]	n	With stenosis n (%) [95%CI]	
Women	53	0 (0%) [NA]	64	8 (12.5%) [4.2-20.8%]	p = 0.008
65-74 years of age†	77	6 (7.8%) [1.7-13.9%]	40	2 (5.0%) [0.0-12.1%]	p = 0.570
Regular dental‡	94	5 (5.3%) [0.7-9.9%]	23	3 (13.0%) [0.0-27.9%]	p = 0.188
Myocardial infarction	24	4 (16.7%) [0.6-32.7%]	93	4 (4.3%) [0.1-8.5%]	p = 0.032
Heart Failure	6	0 (0%) [NA]	111	8 (7.2%) [2.3-12.1%]	p = 0.496
Current angina	17	4 (23.5%) [1.1-46.0%]	100	4 (4.0%) [0.1-7.9%]	p = 0.003
Symptomatic peripheral artery disease	4	2 (50.0%) [0.0-100%]	113	6 (5.3%) [1.1-9.5%]	p = 0.0005
Diabetes	32	3 (9.4%) [0.0-20.1%]	85	5 (5.9%) [0.8-11.0%]	p = 0.505
Hypertension	88	8 (9.1%) [3.0-15.2%]	29	0 (0%) [NA]	p = 0.093
Current smoking	21	4 (19.0%) [0.7-37.4%]	96	4 (4.2%) [0.1-8.2%]	p = 0.014
Blood pressure medicine	85	8 (9.4%) [3.1-15.7%]	32	0 (0%) [NA]	p = 0.072
Lipid lowering medicine	61	8 (13.1%) [4.4-21.8%]	56	0 (0%) [NA]	p = 0.005
Platelet inhibiting or anticoagulant medicine	53	8 (15.1%) [5.1-25.1%]	64	0 (0%) [NA]	p = 0.0013
Any cardiovascular event	44	7 (15.9%) [4.7-27.2%]	73	1 (1.4%) [0.0-4.1%]	p = 0.003
Any cardiovascular risk factor	101	8 (7.9%) [2.6-13.3%]	16	0 (0%) [NA]	p = 0.243
All	117	8 (6.8%) [2.2-11.5%]	-	-	-

Differences between basic and cardiovascular subgroups in participants with calcification in the area of the carotid arteries. Based on persons, not individual neck-sides.

*Chi-2-test

† We detected no difference in mean age between the persons with (68.4 years SD 4.5) and without (66.8 years SD 5.6) carotid stenosis, p = 0.44 (t-test)

‡Regular dental - examinations performed in all practices using panorama, e.g. dental and implant treatment.

The mean age for included persons with calcification in the area of the carotid arteries was higher compared to all other persons examined with panorama without calcifications in the area of the carotid arteries, 66.9 years (SD 5.6) versus 49.8 years (SD 18.1), p < 0.001. The persons with calcification in the area of the carotid arteries had a higher prevalence of all cardiovascular parameters and most differences were statistically significant (Table 3).

**Table 3:** Comparisons between included persons and reference persons

	Included persons n (%)	Reference persons n (%)	Significance*
Women	53 (45.3%)	56 (47.1%)	p = 0.786
65-74 years	77 (65.8%)	79 (66.4%)	p = 0.926
Indication - Regular dental†	94 (80.3%)	100 (84.0%)	p = 0.458
Myocardial infarction	24 (20.5%)	7 (5.9%)	p = 0.0009
Heart Failure	6 (5.1%)	3 (2.5%)	p = 0.296
Current angina	17 (14.5%)	2 (1.7%)	p = 0.0003
Symptomatic peripheral artery disease	4 (3.4%)	1 (0.8%)	p = 0.169
Diabetes	32 (27.4%)	10 (8.4%)	p = 0.00014
Hypertension	88 (75.2%)	54 (45.4%)	p < 0.0001
Current smoking	21 (17.9%)	8 (6.7%)	p = 0.009
Blood pressure medicine	85 (72.6%)	56 (47.1%)	p < 0.0001
Lipid lowering medicine	61 (52.1%)	27 (22.7%)	p < 0.0001
Platelet inhibiting or anticoagulant medicine	53 (45.3%)	34 (28.6%)	p = 0.008
Any cardiovascular event	44 (37.6%)	12 (10.1%)	p < 0.0001
Any cardiovascular risk factor	101 (86.3%)	78 (65.5%)	p = 0.00019
All	117	119	-

Differences in the rate of basic and cardiovascular subgroup findings between included persons with a calcification in the area of the carotid arteries and reference persons.

*Chi-2-test

† Regular dental - examinations performed in all practices using panorama, e.g. dental and implant treatment.

Calcification in the area of the carotid arteries appeared unilaterally in 40 included persons and bilaterally in 77 persons. All nine carotid stenoses had a calcification in the area of the carotid arteries on the corresponding neck side. Five persons had 50-69% stenosis and were managed with medical cardiovascular prevention and re-examination of their carotid stenosis. Three persons had a 70-99% carotid stenosis (one of these also had a contralateral 50-69% stenosis). These persons were offered CEA: one underwent CEA, one refused, and one died before the operation. We detected no carotid occlusions. We detected a calcified atherosclerotic lesion (causing a < 50% stenosis) in 99% (108/109) of the persons with calcifications in the area of the carotid arteries but without a 50-99% carotid stenosis. The time between the panoramic examination and the ultrasound examination was 83 days (SD 54) on average.

## Discussion

The main finding of this study was the high prevalence of significant (50-99%) carotid stenosis in men with incidentally detected calcification in the area of the carotid artery seen on panoramic examinations. Thus, in this subpopulation, directed screening for asymptomatic carotid stenosis is indicated [12].

We missed six out of 123 eligible persons in the study. Thus the inclusion rate of the intended population was good. The kappa values for the existence of a calcification in the area of the carotid arteries on each side of the neck showed good agreement between observers.

We detected a different rate of calcifications in the area of the carotid arteries compared with previous studies [13,14]. This difference has at least three possible explanations: 1) Not all panoramic examinations include the area of the carotid arteries, thus calcifications in the vessels can be missed. 2) For inclusion in the study, we required that the calcification in the area of the carotid arteries seen on panoramic radiograph should be confirmed on APP. A calcification can be undetectable in the APP examination if the spine superimposes the calcification. This may have reduced the number of detected calcifications in the area of the carotid arteries in this study. 3) There are several known risk factors for arterial calcification such as age, dietary factors, and smoking habits that differ among geographic areas [17]. Thus, it is possible that the prevalence of calcifications in the area of the carotid arteries varies between different studies populations.

We detected differences in the proportion of included persons between the three referral subgroups. The 'regular dental' subgroup had the highest rate of inclusion in the study; persons in the 'specialised dental' subgroup were younger (mean age 36.5 years, SD 17.3) and should therefore have a lower degree of atherosclerosis. Thirty of 47 persons with calcification in the area of the carotid arteries in the 'specialised medical' group were excluded; in most cases, these persons were referred for the panoramic radiographs due to a diagnosis of cancer or serious co-morbidity and were excluded since these diagnoses made them ineligible for asymptomatic CEA.

We included a sex- and age-matched reference group in order to determine if the persons with calcification in the area of the carotid arteries have an average or above average burden of atherosclerotic disease. The reference group was representative for age, sex, and cause of referral for the panoramic examination compared to study persons with calcifications in the area of the carotid arteries. Based on the differences seen in Table 3 we believe that persons with calcification in the area of the carotid arteries have an above average burden of atherosclerotic disease. In general, arterial calcification is more prevalent in persons with cardiovascular disease [17]; thus, this finding was expected.

It is uncertain what the lowest degree of carotid stenosis is that entails benefit with asymptomatic CEA. The results from the ACST study suggest that asymptomatic CEA is of similar benefit for persons with 70-99% and with 50-69% carotid stenosis [2]. Ongoing studies might clarify the benefit vs. risk ratio of carotid surgery or stenting in various degrees of asymptomatic carotid stenoses [7]. Due to the present uncertainty in clinical indication, we have presented data for 50-99% carotid stenosis.

Overall, we detected a lower prevalence of carotid stenosis in persons with calcification in the area of the carotid arteries compared with previous studies [13,14]. One reason for this is that we only examined persons eligible for asymptomatic CEA, with age below 75 years [1,2]. Persons > 75 years of age were examined in previously published studies [13,14] and the prevalence of carotid stenosis increases with increasing age [11]. In the largest of the previous studies, 94% of included persons were men [13]; we found men to have a higher prevalence of carotid stenosis.

We found several significant subgroup heterogeneities for the prevalence of 50-99% carotid stenosis. These finding were expected since they mark atherosclerotic disease in other parts of the body. These clinical features are most certainly not independent of each other, for example: persons with vascular events are prescribed the medications analysed. A multivariate analysis to test for independence was not performed since the total number of outcomes - i.e. 50-99% carotid stenosis - was few (n = 8). We advocate that until such a multivariate analysis can be performed in a larger study, only male sex should be used as basis for selection to carotid screening with ultrasound since: (1) it is one of few factors available to dentists; (2) it was one of few factors that was clinically useful, i.e. in addition to be significant, when positive, it included all findings of carotid stenosis.

Age did not affect the prevalence of carotid stenosis in the included population. This could be a false negative finding since the prevalence of carotid stenosis increases with increasing age [11]. However, until shown otherwise, age should not be used as a criterion to go ahead with or abstain from carotid ultrasound screening in persons < 75 years with calcifications in the area of the carotid arteries.

We have not analysed the appearance (intensity, size, and/or shape) of the calcification in the area of the carotid arteries on the panoramic images. These factors might be useful to further select persons for carotid screening.

Our further clinical experience confirms the results presented here: between the study's stop date in February 2009 and February 2011, we have found 65 additional men with calcification in the area of the carotid arteries on panoramic examinations. Carotid ultrasound examinations revealed that six of these individuals had 50-99% carotid stenosis and two have undergone CEA. The carotid ultrasound examinations also revealed an aneurysm (12 mm in diameter) of the internal carotid artery in one person and a carotid occlusion in one person. Our collected experience is that, of 129 men with calcification in the area of the carotid arteries found on panoramic radiographs, 14 (10.9%, 95%CI 5.4-16.3%) had a 50-99% carotid stenosis and 15 (11.6%, 95%CI 6.0-17.2%) had a 50-100% carotid stenosis; significantly over the 5% pre-specified threshold, p = 0.005 and p = 0.002 respectively (Binomial test).

The findings of this study must be interpreted with caution since we did not find a prevalence of carotid stenosis > 5% in the whole study group, but only in subgroups [18]. However, previous similar studies on panoramic examination report the prevalence of 50-99% carotid stenosis to be > 5% [13,14] of persons with calcifications in the area of the carotid arteries; the reproducibility of this finding strengthens this study's finding [18]. Carotid ultrasound was not performed on persons in the reference group. There were too few cases to perform a multivariate subgroup analysis. We did not analyse on the appearance of the calcification in the area of the carotid arteries. This warrants further studies, which we plan to conduct.

The high prevalence of carotid stenosis in the persons with calcifications in the area of the carotid arteries was probably influenced by both the revelation of a local atherosclerotic process and by the above average burden of generalised atherosclerotic disease. To what extent each factor contributed to this finding is unknown.

We only included persons that were eligible for asymptomatic CEA; this mirrors clinical practice. Contrary to the largest previous study, men and women were included in almost equal proportions; however, only men were found to have carotid stenosis. Thus, this study is externally valid for both sexes. There were no significant differences in the prevalence of 50-99% carotid stenosis between the subgroups based on reason for referral for the panoramic examination. The results are valid for dentists in general practice and for centres that perform examinations corresponding to the subgroups 'regular dental' or 'specialised dental'.

The design of this study intended to mirror a clinical practice based from the ACST study [2]; the conclusion was based on a threshold for what prevalence of carotid stenosis is required for carotid screening that was calculated for the same clinical practice [12]. Nowadays, statin treatment is more common than at the time of the ACST study; Recent, ongoing, and coming trials will determine if asymptomatic CEA is still indicated in patients with statin and other cardiovascular preventive treatment, and in whom asymptomatic CEA is of benefit [7-10]. Thus, it is likely that the clinical practice for persons with asymptomatic carotid stenosis will change within a few years. If so, the threshold for what prevalence of carotid stenosis is required for carotid ultrasound screening must be recalculated based on that clinical practice. Our results should then be compared to this new threshold and the conclusions revised if necessary.

## Conclusions

Carotid screening with ultrasound should be performed in men with the incidental finding of calcification in the area of the carotid arteries seen on panoramic examinations, confirmed with an anterio-posterior examination, if they are otherwise eligible for asymptomatic carotid endarterectomy.

## List of abbreviations

APP: Anterio-Posterior Projection; CEA: Carotid EndArterectomy; SD: Standard Deviation; TIA: Transient Ischemic Attack.

## Competing interests

The authors declare that they have no competing interests.

## Authors' contributions

EPJ assisted in the study design, gathered most of the data, analyzed all the data, and wrote the first draft of the manuscript. JA came up with the general study design, gathered some of the data, made constructive comments to the manuscript, and served as mentor for MG. MG gathered some of the data and wrote minor parts of the manuscript. KK was responsible for the carotid ultrasound examinations and made constructive comments to the manuscript. ELJ assisted in the study design, gathered some of the data, made constructive comments to the manuscript, and served as mentor for MG. PW came up with the general study design, made constructive comments to the manuscript, and served as mentor for EPJ. All authors have read and approved the final version of the manuscript.

## Pre-publication history

The pre-publication history for this paper can be accessed here:

http://www.biomedcentral.com/1471-2261/11/44/prepub
